# Persistence with daily growth hormone among children and adolescents with growth hormone deficiency in the UK

**DOI:** 10.3389/fendo.2022.1014743

**Published:** 2022-11-03

**Authors:** Jane Loftus, Jen Wogen, David Oliveri, Darrin Benjumea, Priti Jhingran, Yong Chen, Jose Alvir, Elena Rivero-Sanz, Jack C. Kowalik, Michael P. Wajnrajch

**Affiliations:** ^1^ Value and Evidence, Pfizer Ltd., Tadworth, United Kingdom; ^2^ Epidemiology, Genesis Research, Hoboken, NJ, United States; ^3^ Real World Evidence Analytics, Genesis Research, Hoboken, NJ, United States; ^4^ Evidence Strategy, Genesis Research, Hoboken, NJ, United States; ^5^ Real World Evidence, Pfizer Inc., Collegeville, PA, United States; ^6^ Biostatistics, Pfizer Inc., New York, NY, United States; ^7^ UK Medical Affairs, Pfizer Ltd., Tadworth, United Kingdom; ^8^ UK Health & Value, Pfizer Ltd., Tadworth, United Kingdom; ^9^ Global Medical Affairs, Pfizer Inc., New York, NY, United States; ^10^ Grossman School of Medicine, New York University, New York, NY, United States

**Keywords:** persistence, growth hormone deficiency, children, discontinuation, United Kingdom

## Abstract

**Background:**

Children with growth hormone deficiency (GHD) are treated with daily somatropin injections; however, poor treatment persistence and adherence have been recognized previously and have been shown to negatively impact growth outcomes. A recent real-world study of a US pediatric GHD population found that a substantial proportion of children discontinued somatropin therapy, but similar data for a real-world UK population are lacking.

**Objectives:**

To describe the discontinuation of, and persistence with, daily somatropin treatment among children with GHD in the UK.

**Methods:**

This was a retrospective cohort study of children (≥3 and <16 years old) with ≥1 medication prescription for daily injectable somatropin from 1 July 2000 to 31 December 2020 in the IQVIA Medical Research DATA (IMRD) database. Early persistence was defined as the proportion of children prescribed ≥1 somatropin refill (≥2 prescriptions). Discontinuation was defined as the first date at which a medication gap for somatropin (of >60 or >90 days between prescriptions) occurred. Kaplan–Meier methods were used to evaluate persistence (non-discontinuation) over time to assess time to first discontinuation event. Cox proportional hazards models were used to evaluate the relationship between patient characteristics and time to medication discontinuation.

**Results:**

Among the cohort identified in this study (*n* = 117), the majority (*n* = 84, 71.8%) had 48 months of available follow-up; 56.4% were boys and the mean (median) age was 8.6 (8.0) years. About 98% exhibited early persistence, but persistence over the follow-up period decreased with follow-up duration. Using the conservative 90-day gap definition of persistence, an estimated 72.4%, 52.8%, and 43.3% were persistent at 12, 36, and 48 months. Lower persistence rates were observed using the 60-day definition. No significant patient predictors of time to discontinuation were identified.

**Conclusions:**

Despite high early persistence with somatropin, a high percentage of children with GHD were increasingly non-persistent over time. More than 1 in 4 were non-persistent at 12 months and more than 1 in 2 were non-persistent at 48 months of follow-up. These results suggest that strategies to support improved medication-taking behavior among children with GHD in the UK are warranted.

## Introduction

Pediatric growth hormone deficiency (pGHD) affects an estimated 1 in 3,500–4,000 children in the United Kingdom (UK) (1). Recombinant human growth hormone (r-hGH; somatropin) became available in the UK in 1988 and remains the standard of care for pGHD. Treatment is administered daily *via* subcutaneous injections, and there are currently seven preparations of somatropin licensed in the UK (1). The goal of treatment is to improve the growth rate in childhood to achieve a final adult height within 2 standard deviations of the population mean and prevent or improve metabolic impairments associated with GHD.

In the UK, the recommended starting dose of somatropin is 0.16 to 0.24 mg/kg/week divided into six or seven daily subcutaneous injections (1, 2). Subsequent dosing varies and should be individualized to the patient based on insulin-like growth factor 1 (IGF-1) concentrations, a mediator of various growth-promoting functions of the growth hormone (GH) (2). Guidelines differ on the optimal frequency of patient monitoring after treatment initiation, with the general consensus landing between once every 3–6 months (1–3). Discontinuation of somatropin should only occur under one of the following circumstances: 1) growth velocity within the first year of treatment increases <50% from baseline, 2) the final height of the patient is approached and growth velocity is <2 cm of total growth in 1 year, 3) there are insurmountable problems with adherence, or 4) final height is attained (1, 2).

Suboptimal adherence to somatropin has been well documented in many populations and associated with a negative impact on growth response (4–6). UK-based studies have also demonstrated the association between adherence to treatment and improved growth outcomes. Almost 1 in 4 (23%) children with pGHD (*n* = 75) studied from regional UK pediatric endocrinology clinics missed >2 injections per week, which was associated with lower predicted height velocities (7). In another UK-based study (*n* = 52), year-on-year height standard deviation scores were significantly increased for children who were adherent (defined as the proportion of days covered >0.80) to treatment over 3 years, but there was no significant increase for children who were non-adherent (8). Although the association between GH persistence and growth outcomes has been less frequently evaluated than adherence, one US study of children with pGHD found better treatment persistence (continuing with treatment) to be associated with improved height outcomes (9).

Prior research has estimated discontinuation rates of ~4%–42% and persistence duration of 2.1–3.6 years using different methods and study populations (10–13). Non-persistence and discontinuation may be higher among adolescents than children; one study found non-persistence of 22% and 15%, with discontinuation of 22% and 15% among adolescents and children, respectively (10). To date, there have been no real-world studies of somatropin discontinuation or persistence and associated predictors among a pGHD population in the UK. This study aimed to describe the discontinuation of, and persistence to, daily somatropin treatment among children with GHD in the UK.

## Materials and methods

### Data source

This was a retrospective cohort study utilizing the IQVIA Medical Research DATA (IMRD) database, which contains longitudinal non-identified primary care electronic health records (EHR) from 832 UK practices representing more than 19 million patients in the UK (~27% of the population). The IMRD incorporates data from The Health Improvement Network (THIN), a Cegedim Database, where data are generated from the daily record keeping of GPs and other clinical staff within the practice.

### Study population

Children with ≥1 prescription claim for somatropin, >1 diagnosis code for pGHD in the baseline period 6 months prior to or on the index date, and aged between 3 and <16 years at the time of the index date were included in this analysis. The date of the first prescription for somatropin during the identification period (1 July 2000 to 31 December 2020) was defined as the index date. Continuous enrollment in the database during the 6-month baseline period pre-index date (baseline period) was also required. Exclusions included prescription records for somatropin during the 6-month baseline period pre-index date, or other causes/diagnoses associated with short stature, i.e., psychosocial dwarfism, celiac disease, uncontrolled primary hypothyroidism, or rickets any time prior to the index date.

Four non-exclusive patient cohorts, based on the duration of continuous follow-up, were examined to assess discontinuation and persistence longitudinally: 3+ months (cohort A; at least 3 and up to 48 months of follow-up), 12 months (cohort B), 36 months (cohort C), and 48 months (cohort D) of follow-up.

### Study measures

Study measures (i.e., persistence and discontinuation) were evaluated throughout follow-up (up to 48 months). Early persistence was defined as children with ≥1 refill of somatropin (≥2 prescriptions). Discontinuation was defined as the first observation of a gap of >60 or >90 days between successive somatropin prescription fill dates, two standard metrics used in adherence and persistence studies. For the purposes of this study, the 90-day discontinuation gap is the primary definition of discontinuation to reflect the recommended monitoring patterns of patients on somatropin (2, 3). The date of the last prescription which occurred at the beginning of the qualifying observed gap was defined as the discontinuation date, and the time to discontinuation was calculated as [discontinuation date − index date]. Persistence was defined as continuous refills of somatropin (no discontinuation, i.e., gaps >60 and >90 days between prescription dates) during the study follow-up period.

Other study variables, including baseline demographics and clinical characteristics, were also evaluated. Demographics included patient sex and age. Clinical characteristics assessed during the baseline period included the number of concomitant medications, using available drug classification information. Baseline mental health diagnoses were also assessed, which included attention deficit conduct and disruptive behavior disorders and anxiety/depression and related mental health disorders. The total number of all-cause healthcare visits per patient during follow-up was also calculated.

### Statistical analysis

Descriptive analyses were performed for all study variables. Means and standard deviation (SD) were calculated for all continuous variables. Frequency counts and percentages were calculated for categorical variables. Persistence (non-discontinuation) over time was evaluated using Kaplan–Meier methods to assess time to first discontinuation event and proportion persistent at each time point among children with 3+ months (cohort A) of follow-up. Associations between patient demographic and clinical characteristics and time to medication discontinuation were evaluated using Cox proportional hazards models. Hazard ratios with 95% confidence intervals (CIs) and *P*-values were calculated for each of the characteristics.

## Results

### Cohort identification

**Figure 1:**
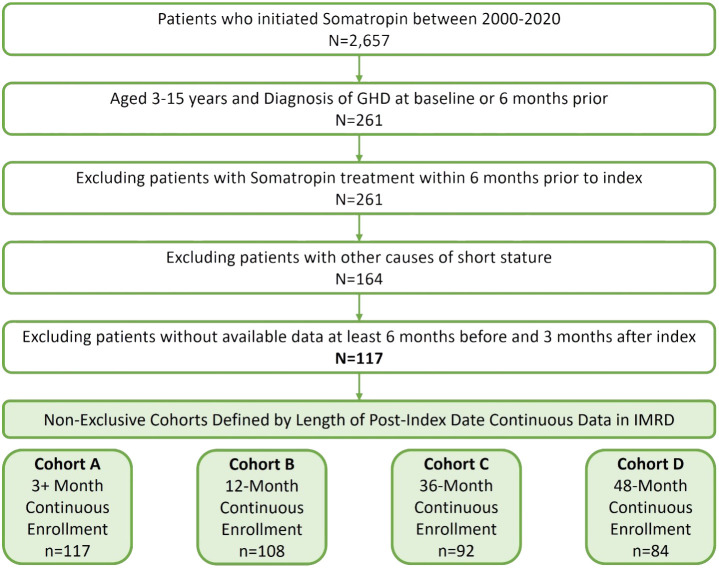
Cohort sample identification of children (≥3 and **<**16 years) with pGHD treated with daily somatropin in the UK.

There were 2,657 children identified who were initiated on somatropin treatment between 1 July 2000 and 31 December 2020 (**Figure 1**). Approximately 10% (261) of these children were aged ≥3 and <16 years old with a diagnosis of GHD at baseline or in the 6 months prior. Due to other causes/diagnoses associated with short stature, 97 children were excluded. Children were required to have data available in IMRD for at least 6 months prior and 3 months post index. The final study analysis included 117 children in four non-exclusive cohorts defined by the available follow-up after the index date, 3+ months (cohort A, *n* = 117), 12 months (cohort B, *n* = 108), 36 months (cohort C, *n* = 92), and 48 months (cohort D, *n* = 84).

### Cohort demographic and clinical characteristics

For each cohort, demographic and clinical characteristics are outlined in **Table 1**. Across all cohorts, 56.4% of children were male patients and the mean (median) age was 8.6 (8.0) years. The majority of children (*n* = 84, 71.8%) had 48 months of follow-up. The mean number of concomitant medications at baseline was 2.8–3.3. At baseline, 15.4%–17.9% of children had any mental health diagnosis. Anxiety/depression and related mental health disorders were more prevalent among the pGHD cohorts than attention deficit conduct and disruptive behavior disorders. Across cohorts A–D, 10.3%–12.0% of children had anxiety/depression and related mental health disorders, and 5.6%–7.1% of children had attention deficit conduct and disruptive behavior disorders.

**Table 1:** Baseline demographic and clinical characteristics of children (≥3 and **<**16 years) with pGHD treated with daily somatropin in the UK.

	Cohort A: 3+ months of follow-up (*n* = 117)	Cohort B: 12 months of follow-up (*n* = 108)	Cohort C: 36 months of follow-up (*n* = 92)	Cohort D: 48 months of follow-up (*n* = 84)
Sex, *n* (%)				
Female	51 (43.6%)	46 (42.6%)	38 (41.3%)	33 (39.3%)
Male	66 (56.4%)	62 (57.4%)	54 (58.7%)	51 (60.7%)
Age (continuous)				
Mean (SD)	8.62 (3.32)	8.56 (3.34)	8.67 (3.33)	8.73 (3.31)
Median (Q1–Q3)	8 (6.00–11.00)	8 (6.00–11.00)	8 (6.00–11.50)	8 (6.00–11.50)
Age (categorical), *n* (%)				
3–7 years	48 (41.0%)	46 (42.6%)	36 (39.1%)	32 (38.1%)
8–11 years	41 (35.0%)	36 (33.3%)	33 (35.9%)	31 (36.9%)
12–<16 years	28 (23.9%)	26 (24.1%)	23 (25.0%)	21 (25.0%)
Number of concomitant medications 6 months at baseline				
Mean (SD)	2.80 (3.83)	3.00 (3.91)	3.27 (4.11)	3.24 (3.99)
Median (Q1–Q3)	1.00 (0.00–4.00)	2.00 (0.00–4.00)	2.00 (0.50–4.00)	2.00 (0.00–4.00)
Baseline mental health (MH) diagnoses, *n* (%)				
Any baseline MH diagnosis^a^	18 (15.4%)	17 (15.7%)	16 (17.4%)	15 (17.9%)
Attention deficit conduct and disruptive behavior disorders	7 (6.0%)	6 (5.6%)	6 (6.5%)	6 (7.1%)
Anxiety/depression and related MH disorders	12 (10.3%)	12 (11.1%)	11 (12.0%)	10 (11.9%)

a Any baseline mental health diagnosis is defined as a diagnosis of attention deficit conduct and disruptive behavior disorders and/or anxiety/depression and related mental health disorders.

### Medication persistence and discontinuation

**Figure 2:**
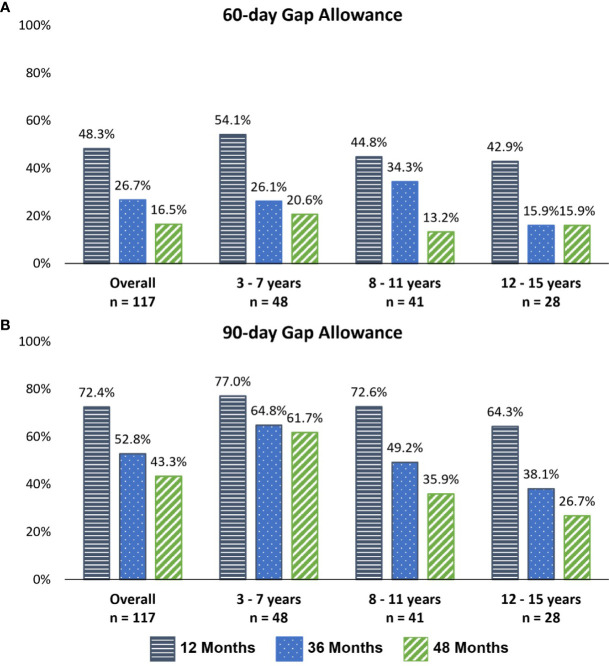
Persistence with somatropin throughout follow-up, by age group and follow-up cohort. **(A)** A 60-day gap definition of discontinuation. **(B)** A 90-day gap definition of discontinuation.

Early persistence, defined as >1 refill of somatropin (≥2 prescriptions), was very high at 98% across all children (**Table 2**). The mean time between refills was 35.8, 40.9, and 41.7 days for the 12-, 36-, and 48-month follow-up cohorts. Using the 90-day definition for discontinuation, the mean number of fills prior to discontinuation was 11.7, 27.8, and 34.0 mean fills for the 12-, 36-, and 48-month follow-up cohorts, respectively, whereas using the shorter 60-day gap definition for discontinuation, the mean number of fills prior to discontinuation was 10.2, 21.6, and 24.5 for over the same follow-up intervals. Persistence was lower among older children (8–11 and 12–15 years) than young children (3–11 years) at 12, 36, and 48 months (**Figure 2**). Persistence over the follow-up period decreased with follow-up duration, based on the definition of discontinuation (60- or 90-day gap). Using the 90-day gap definition, among children with ≥3 months of follow-up, persistence at 12, 36, and 48 months was 72.4%, 52.8%, and 43.3%, and using the 60-day gap definition, it was 48.3%, 26.7%, and 16.5% at 12, 36, and 48 months, respectively.

**Table 2:** Medication utilization and number of healthcare visits among children (≥3 and **<**16 years) with pGHD treated with daily somatropin in the UK.

	Cohort B: 12 months of follow-up (*n* = 108)	Cohort C: 36 months of follow-up (*n* = 92)	Cohort D: 48 months of follow-up (*n* = 84)
Early persistence status			
Early persistence	106 (98.1%)	90 (97.8%)	83 (98.8%)
Number of fills			
Mean (SD)	12.2 (5.21)	31.66 (14.6)	40.31 (18.41)
Median (Q1–Q3)	12 (8.50–15.50)	32 (20.50–42.00)	40 (27.00–52.00)
Time (days) between refills			
Mean (SD)	35.76 (17.53)	40.89 (26.52)	41.74 (28.87)
Median (Q1–Q3)	30.3 (23.90–42.00)	30.8 (24.40–46.80)	31.6 (26.80–45.50)
60-day gap allowance			
Number of fills, prior to discontinuation			
Mean (SD)	10.16 (6.86)	21.57 (19.46)	24.52 (24)
Median (Q1–Q3)	10.5 (3.00–15.00)	16 (3.00–38.50)	16 (3.50–43.00)
Time (days) between refills, prior to discontinuation			
Mean (SD)	27.29 (10.4)	27.15 (10.45)	27.41 (10.12)
Median (Q1–Q3)	25.8 (21.30–32.50)	26 (20.60–32.30)	26 (20.70–32.00)
90-day gap allowance			
Number of fills, prior to discontinuation			
Mean (SD)	11.70 (5.85)	27.75 (17.93)	33.98 (23.17)
Median (Q1–Q3)	12 (8.00–15.50)	29 (9.00–41.50)	37 (9.00–50.50)
Time (days) between refills, prior to discontinuation			
Mean (SD)	31.05 (13.19)	31.93 (13.68)	32.29 (12.48)
Median (Q1–Q3)	28 (22.40–37.00)	29 (22.60–38.50)	30 (24.00–37.90)
Visit frequency			
Number of all-cause visits during follow-up			
Mean (SD)	44.05 (26.39)	115.12 (64.47)	151.29 (85.57)
Median (Q1–Q3)	38 (26.00–53.00)	97 (68.00–148.00)	119.5 (92.50–180.00)

**Figure 3:**
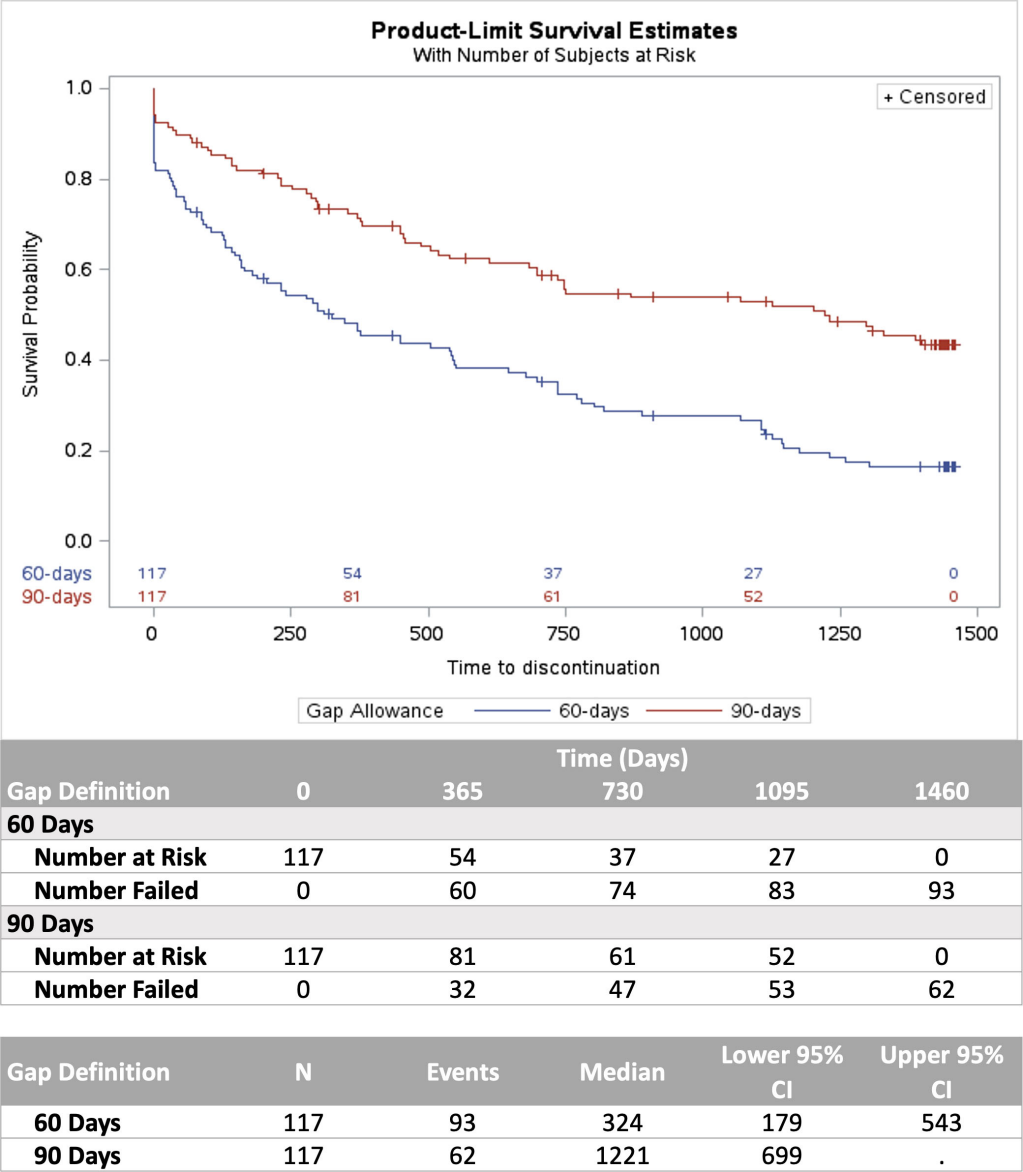
Kaplan–Meier analysis of time to discontinuation using 60- and 90-day gap definitions.

Among the 3+-month follow-up cohort (A) (up to 48 months of follow-up), time to GH discontinuation is depicted *via* Kaplan–Meier curves for both the 60- and 90-day definitions of discontinuation in **Figure 3**. The estimated median time to discontinuation was 324 (95% CI 179, 543) and 1,221 (95% CI 699, NR) days for the 60- and 90-day definitions of discontinuation, respectively.

Persistence was lower among older children (90-day gap definition) (**Figure 2**). For the 12-month follow-up cohort (B), 87.0% of 3–7-year-old children were persistent, falling to 80.6% among 8–11-year-olds and 69.2% among 12–15-year-olds. For the 48-month follow-up cohort (D), 59.4% of the 3–7-year-old group was persistent, dropping to 45.2% and 38.1% among 8–11- and 12–15-year-old children, respectively.

No patient-level characteristics were significantly associated with time to discontinuation (**Table 3**).

**Table 3:** Cox proportional hazards model, 3+ months of follow-up (cohort A).

Covariate	60-day gap allowance		90-day gap allowance	
	Odds ratio (95% CI)		Odds ratio (95% CI)	
	Unadjusted	Adjusted	Unadjusted	Adjusted
Age				
3–7 years	0.86 (0.53, 1.37)	0.77 (0.48, 1.25)	0.81 (0.51, 1.29)	0.67 (0.40, 1.10)
8–11 years	0.72 (0.44, 1.17)	0.57 (0.34, 0.96)	0.80 (0.49, 1.3)	0.66 (0.39, 1.11)
12–<16 years	Reference group	Reference group	Reference group	Reference group
Sex				
Male	0.93 (0.64, 1.36)	0.81 (0.55, 1.20)	0.86 (0.59, 1.26)	0.79 (0.53, 1.17)
Female	Reference group	Reference group	Reference group	Reference group
Concomitant medications				
0	Reference group	Reference group	Reference group	Reference group
1	0.64 (0.37, 1.10)	0.66 (0.38, 1.14)	0.86 (0.50, 1.48)	0.85 (0.49, 1.47)
2	1.58 (0.84, 2.99)	1.67 (0.88, 3.18)	1.4 (0.74, 2.66)	1.34 (0.70, 2.57)
≥3	0.56 (0.36, 0.88)	0.49 (0.30, 0.80)	0.74 (0.47, 1.14)	0.63 (0.39, 1.01)
Mental health				
Mental health comorbidity: yes	0.81 (0.48, 1.36)	0.94 (0.55, 1.59)	0.72 (0.43, 1.21)	0.69 (0.40, 1.18)
Mental health comorbidity: no	Reference group	Reference group	Reference group	Reference group
Attention deficit conduct: yes	1.02 (0.48, 2.21)		0.76 (0.35, 1.65)	
Attention deficit conduct: no	Reference group		Reference group	
Anxiety/depression: yes	0.74 (0.39, 1.38)		0.73 (0.39, 1.36)	
Anxiety/depression: no	Reference group		Reference group	

## Discussion

This retrospective study of children with pGHD found high levels of discontinuation, despite very high levels of early persistence. At 48 months (cohort D, *n* = 86), more than 1 in 2 (>50%) patients discontinued therapy using a conservative 90-day gap definition. Using a shorter day gap definition of 60 days, more than 4 in 5 (>80%) discontinued therapy over the same timeframe. The differences between the 90- and 60-day gap definition may suggest that the high discontinuation rates using the 60-day definition may be misleading or an overestimation of discontinuation as a large proportion of patients did continue therapy between the 60- and 90-day cutoff periods. In line with previously published studies, discontinuation increased over follow-up time and with increasing child age. This observation may be attributed to the approach or attainment of final height or sufficiently slowed growth velocity as children age to warrant somatropin discontinuation per guideline recommendations (2, 3).

A previous study using a self-reporting survey also found higher rates, among adolescents than children, of non-persistence (22% and 15%) and discontinuation (11% and 5%) (10). Persistence studied using the Easypod autoinjector found median persistence with treatment to be 2.1 years (11). A retrospective database study of children initiating GH treatment in Israel found mean persistence of 3.6 ± 1.6 years, and 3.7% of children discontinued (gap of 365 years without treatment) (12). A recent US-based study found that 42.2% of pGHD children discontinued somatropin therapy (gap of >60 days without treatment) within 48 months of follow-up (13).

Miller et al. found that US children from the American Norditropin Studies: Web-enabled Research (ANSWER) registry who achieved final height (*n* = 288) took somatropin longer (46 ± 21 months, *n* = 288) than those who discontinued for any reason (9). Suboptimal adherence to somatropin daily injections (usually defined as missing >1 dose per week) has been shown to lead to poorer growth outcomes (4, 5). A New Zealand-based study, using the rate of returned vials, found that children with good compliance (defined as no more than one missed dose per week) had significantly greater linear growth (5). A retrospective cohort study of US claims data showed that adherent children grew an additional 1.8 cm over 1 year of treatment than non-adherent children (6). A UK-based study of 75 pGHD children who attended regional pediatric endocrinology clinics found that almost 1 in 4 (23%) missed >2 injections per week and this was associated with lower predicted height velocities (7). In another UK-based study of 52 children, year-on-year height SDS was significantly increased for children who were adherent (defined as the proportion of days covered >0.80) to treatment over 3 years, but there was no significant increase for children who were non-adherent (8).

The present study did not find any factors which increased the risk of discontinuation, which have been found in other studies. At baseline, the mean number of concomitant medications was 2.8–3.3. At baseline, 15.4%–17.9% of children had any mental health diagnosis with anxiety/depression and related mental health disorders more prevalent than attention deficit conduct and disruptive behavior disorders. Neither variable was a significant predictor of discontinuation. A recent study using the Easypod autoinjector found indicators with a positive impact on persistence including at least one dose change per year, starting treatment at an early age, high adherence (≥85%), customized injection speed setting, and male sex (11). The same study found variations in persistence across different regions—1.0 years in the Asia-Pacific, 1.5 years in North America, and 2.8 years in Europe (11). Several previous studies have found age to be a predictor of somatropin adherence (5, 14–16).

It is well acknowledged that a severe limitation to the efficacy of GH in patients with pGHD is adherence to daily injections (17). Long-acting GH preparations (LAGH), injected once weekly as opposed to once daily, have recently been approved for use in the US, EU, Japan, Canada, and Australia and are believed to be a potential solution for improvements in patient adherence (18). Although no published data have emerged on the impact of LAGH on adherence from real-world use, a recently published clinical trial demonstrated that treatment burden, as evaluated by life interference, was significantly reduced when patients were treated with somatrogon once weekly compared to once daily Genotropin, and the overall treatment experience for somatrogon was preferred by patients and caregivers (19). Another recent study found a preference for LAGH and reduced treatment burden in patients that switched from daily somatropin to weekly lonapegsomatropin (20). A reduced treatment burden and preference for a LAGH should translate into improved adherence and outcomes in the real world; prospective real-world studies which have recently been initiated will aim to evaluate this (21, 22).

### Limitations

This was a retrospective study of longitudinal non-identified EHR data collected by a cohort of GPs during regular primary care visits; therefore, only data required for the management of patient care were collected, which may have led to missing data and bias. Also, adherence to treatment could not be calculated as the days’ supply of prescribed somatropin was not accurately captured in the IMRD. Additional research using different sources of data, including secondary data sources with more detailed information on medication dispensed, may provide additional context for understanding patient adherence and persistence and associated predictors across different patient populations. The final cohort included only 117 pGHD children, and thus, a small sample size limits the statistical power of the study. This study was specific to children in the UK, which has socialized healthcare, and therefore, the conclusions could be relevant to EU countries but may not be generalizable to other populations. Finally, as we were unable to assess the reasons for somatropin discontinuation, we could not determine the proportion of children who may have been directed by the treating physician to end treatment with GH treatment.

## Conclusion

This study found high levels of somatropin discontinuation across age groups, and as with previous studies, discontinuation increases over time. Poor treatment persistence can negatively affect growth among pGHD children; therefore, strategies to improve treatment persistence among pGHD children may improve clinical outcomes. The recently introduced long-acting GH preparations, which are given once weekly as opposed to once daily, have the potential to improve adherence and persistence, and this may also lead to improved outcomes. Additional research is warranted among larger study samples, different patient populations, and using different secondary data sources (such as pharmacy claims data) to quantify non-persistence to somatropin, further characterize the potential impact on growth outcomes, and inform future strategies to address discontinuation.

## Data availability statement

Publicly available datasets were analyzed in this study. This data can be found here: Database leveraged in this study is the UK EMR IQVIA Medical Research Data (IMRD). For more information: https://www.iqvia.com/library/fact-sheets/uk-emr-iqvia-medical-research-data.

## Author contributions

JL, JW, DB, PJ, YC, JA, ES, JK, and MW contributed to the conception and design of the study. DO organized the database and performed the statistical analysis. JW with the support of Amy Glenwright from Genesis Research wrote the first draft of the manuscript. JL and DB contributed to the writing of the sections of the manuscript. All authors contributed to manuscript revision and read and approved the submitted version. All authors agree to be accountable for all aspects of the study and manuscript in ensuring that questions related to the accuracy or integrity of any part of the study and manuscript are appropriately investigated and resolved.

## Funding

This study was sponsored by Pfizer.

## Acknowledgments

Medical writing support was provided by Amy Glenwright at Genesis Research and was funded by Pfizer.

## Conflict of interest

JL, YC, JA, ES, JK, and MW are all employees of Pfizer Inc. and may hold stock/stock options. JW, DO, DB, and PJ are all employees of Genesis Research, LC, which was a paid consultant to Pfizer for this study.

The authors declare that this study received funding from Pfizer Inc. The funder had the following involvement with the study: study design, data collection and analysis, decision to publish, and preparation of the manuscript.

## Publisher’s note

All claims expressed in this article are solely those of the authors and do not necessarily represent those of their affiliated organizations, or those of the publisher, the editors and the reviewers. Any product that may be evaluated in this article, or claim that may be made by its manufacturer, is not guaranteed or endorsed by the publisher.
